# A prospective cohort study of stroke characteristics, care, and mortality in a hospital stroke registry in Vietnam

**DOI:** 10.1186/1471-2377-12-150

**Published:** 2012-12-03

**Authors:** David L Tirschwell, Thanh G N Ton, Kiet A Ly, Quang Van Ngo, Tung T Vo, Chien Hung Pham, William T Longstreth, Annette L Fitzpatrick

**Affiliations:** 1 Department of Neurology, Harborview Medical Center, Room 3EH70, 325 Ninth Avenue, Seattle, WA, 98104, USA; 2 Department of Dental Public Health Sciences, University of Washington, 1959 NE Pacific Street, Box 356365, Seattle, WA, 98195, USA; 3 Department of Health, 103 Hung Vuong Street, Da Nang, Vietnam; 4 Department of Epidemiology, University of Washington, 1959 NE Pacific Street, Box 359236, Seattle, WA, 98195, USA; 5 Department of Global Health, Harborview Medical Center, 325 9th Ninth, Seattle, WA, 98104, USA

**Keywords:** Hemorrhage, Ischemia, Mortality, Risk factors, Stroke

## Abstract

**Background:**

As low and middle-income countries such as Vietnam experience the health transition from infectious to chronic diseases, the morbidity and mortality from stroke will rise. In line with the recommendation of the Institute of Medicine’s report on “Promoting Cardiovascular Health in the Developing World” to “improve local data”, we sought to investigate patient characteristics and clinical predictors of mortality among stroke inpatients at Da Nang Hospital in Vietnam.

**Methods:**

A stroke registry was developed and implemented at Da Nang Hospital utilizing the World Health Organization’s Stroke STEPS instrument for data collection.

**Results:**

754 patients were hospitalized for stroke from March 2010 through February 2011 and admitted to either the intensive care unit or cardiology ward. Mean age was 65 years, and 39% were female. Nearly 50% of strokes were hemorrhagic. At 28-day follow-up, 51.0% of patients with hemorrhagic stroke died whereas 20.3% of patients with ischemic stroke died. A number of factors were independently associated with 28-day mortality; the two strongest independent predictors were depressed level of consciousness on presentation and hemorrhagic stroke type. While virtually all patients completed a CT during the admission, evidence-based processes of care such as anti-thrombotic therapy and carotid ultrasound for ischemic stroke patients were underutilized.

**Conclusions:**

This cohort study highlights the high mortality due in part to the large proportion of hemorrhagic strokes in Vietnam. Lack of hypertension awareness and standards of care exacerbated clinical outcomes. Numerous opportunities for simple, inexpensive interventions to improve outcomes or reduce recurrent stroke have been identified.

## Background

As low and middle-income countries, such as Vietnam, experience the health transition to chronic diseases, the morbidity and mortality from stroke will rise [1]. By 2030, non-communicable diseases will contribute to three quarters of all deaths worldwide [2]. Over the past four decades, age-adjusted population-based stroke incidence rates in high-income countries decreased by 42% whereas the rates in low and middle-income countries increased more than 100%, constituting an epidemic [3]. Additionally, of the estimated 5.7 million global stroke deaths in 2005, 87% occurred in low and middle-income countries [4]. Using World Bank classification of national income levels, low-income countries have been shown to have significantly higher age and gender-adjusted stroke mortality rates than high-income countries [5]. One of the recommendations of the Institute of Medicine’s report on “Promoting Cardiovascular Health in the Developing World” is to “improve local data” to assess more accurately the burden of disease and determine approaches to mitigation [6]. As an initial phase of projects with the long-term goal of reducing the burden of stroke in Vietnam, we sought to investigate patient characteristics and clinical predictors of 28-day mortality among patients hospitalized for stroke at Da Nang Hospital in Vietnam.

## Methods

Da Nang city is located in central Vietnam, approximately equidistant between Hanoi to the north and Ho Chi Minh City to the south. Da Nang covers approximately 1,255 square kilometers with a population of 890,500 residents (2009 census). In a collaboration between the Da Nang Department of Health and the University of Washington, Seattle, a stroke registry was developed and implemented at Da Nang Hospital, the largest tertiary care center in the region, utilizing the World Health Organization’s Stroke STEPS instrument for data collection [7]. Over one year starting March 1, 2010, all patients diagnosed with a WHO-defined stroke were entered into the registry. The standard WHO definition of stroke was “a focal, or at times global, neurological impairment of sudden onset, and lasting more than 24 h or leading to death, and of presumed vascular origin” [7]. Physicians and nurses from the intensive care unit (ICU), cardiology ward, and general internal medicine ward, who are the traditional caregivers for stroke in Da Nang Hospital, were trained with the data collection instrument and collected patient information including demographic characteristics, clinical characteristics, risk factor information, functional status as measured by the modified Rankin Scale (mRS), in-hospital services, and discharge status. In instances where patients were unable to respond to questions regarding risk factors such as smoking, family members served as proxy respondents. Stroke types were differentiated based on computed tomography (CT) scan interpretations into ischemic stroke, intracerebral hemorrhage (ICH) or subarachnoid hemorrhage (SAH). Patients or their family were contacted at 28 days after stroke by personnel trained to use the WHO data collection instrument to obtain information on vital status and physical function; personnel were not blinded to other clinical data. Data were collected on hardcopy and entered into an electronic database. Institutional review boards in the Da Nang Department of Health and the University of Washington approved all study protocols. All participants or their family members provided informed consent.

### Statistical approach

Because 28-day mortality is a common outcome in our population (36.4%) and because odds ratios provide biased estimates of relative risks (RRs) when outcomes are common [8], we used relative risk regression instead of logistic regression to obtain RRs of death associated with risk factors, demographic and clinical characteristics. We conducted relative risk regression using generalized linear models by specifying a log link, a Poisson distribution for the variance, and robust standard errors to fit our models [9].

Demographic and clinical characteristics were missing to various degrees. Values were missing for prior mRS (n=1), disturbed consciousness (n=1), weakness (n=2), atrial fibrillation (n=88), smoking status (n=16), diabetes (n=126), hypercholesterolemia (n=305), systolic blood pressure (n=3), diastolic blood pressure (n=3), glucose level (n=16), stroke type (n=60) and mortality (n=37). Rather than drop subjects from the analysis, we multiply imputed missing values using the method of imputing by chained equations [2]. This process resulted in 10 datasets with imputed values for variables with missing values. The imputation model included age, sex, education, ward, stroke type, mRS scores (prior, at discharge, at follow-up), risk factors (hypercholesterolemia, atrial fibrillation, smoking status, diabetes), occupational therapy, systolic and diastolic blood pressure, cholesterol level, glucose level, and mortality. The main analyses were then run in parallel on 10 datasets that differed only in their imputed values. The 10 sets of results were combined using standard formulas according to Rubin and Schenker [10]. We obtained both univariate and multivariate estimates of association and corresponding 95% confidence intervals (CI’s), adjusted for the 10 datasets, from our generalized models. In univariate analyses, we presented the average percentages across 10 datasets and the P-value of the Wald test for the unadjusted beta coefficient of each dichotomous and continuous variable in univariate regression models. For variables with more than 2 categories, we presented the P-value for the F-test of the univariate model with n-1° of freedom where n=number of categories. For continuous variables, we used linear regression to compare means across two groups. We conducted the trend test for variables with more than two ordinal categories by including the continuous form of the variable in regression models. As a secondary analysis to determine the effect of using multiple imputation techniques on our results, we also analyzed our data using complete case analysis whereby observations with missing values are dropped.

In an effort to assess utilization of stroke processes of care and because these processes were more likely appropriate in patients who survived until discharge and were not discharged home to die, we restricted our analyses of these processes to this subgroup of patients.

For multivariate analyses, we identified independent predictors of 28-day mortality in regression models using a backward stepwise approach. All predictors of interest were placed into the full model and removed sequentially if corresponding *P*-value of the Wald test exceeded 0.05. Predictors of interest in the initial backward model included characteristics at presentation including age, education, stroke type, systolic and diastolic blood pressure, glucose level, prior stroke, prior mRS, disturbed consciousness, weakness, speech disturbance, and risk factors (atrial fibrillation, smoking status, diabetes). We did not include variables that described the processes of care primarily provided to those who remained alive in the hospital due to likelihood of confounding by indication. All tests were two-sided, and statistical significance was defined as *P*-value < 0.05. All analyses were conducted in Stata 11.1 (College Station, TX).

## Results

A total of 754 patients admitted to Da Nang Hospital with stroke were identified over 1 year beginning March 1, 2010. Mean age of stroke patients was 65.0 (SD=14.8) years, and 293 (38.9%) were female. Stroke type was classified using results of CT scans provided on almost all (above 99%) of patients presenting with stroke symptoms at Da Nang Hospital. A total of 328 (43.5%) were ischemic, 356 (47.2%) were ICH, 10 (1.3%) were SAH and 60 (8.0%) were unspecified. Due to the small number of patients with SAH, we combined ICH and SAH into one category defined as hemorrhagic stroke.

After data were multiply imputed, we observed differences between patients with ischemic stroke and those with hemorrhagic stroke (Table 1). Demographics associated with ischemic stroke were older age and retired or unemployed status. Risk factors associated with ischemic stroke included atrial fibrillation, lower prevalence of hypertension (though hypertension was highly prevalent in both stroke types; ischemic 94.5% vs. hemorrhagic 98.0%) and previous history of stroke. Pre-stroke mRS suggested slightly more baseline disability in the hemorrhagic stroke group. At initial clinical presentation, ischemic stroke patients were less likely to have disturbed consciousness and speech disturbances but more likely to have observed weakness, had lower mean systolic and diastolic blood pressures and higher mean total cholesterol levels.

**Table 1:** Characteristics of 754 stroke patients admitted to Da Nang Hospital according stroke type

**DEMOGRAPHICS**	**Ischemic**	**Hemorrhagic**	p-value*	**Trend p-value**
N (%)	328	366	-	-
Male, %	58.1	64.1	0.13	
Age, years, %			<0.001	<0.001
1^st^ Quartile (31–53)	15.7	31.2		
2^nd^ Quartile (53–65)	23.7	27.7		
3^rd^ Quartile (66–76)	29.3	19.7		
4^th^ Quartile (77+)	31.3	21.4		
Age, years, mean	68.3	61.9		<0.001
Education, %			0.5	0.2
<Primary school	34.4	30.9		
Completed primary and secondary school	46.7	46.4		
≥High school	18.8	22.7		
Employment, %			<0.001	
Currently employed	26.7	49.4		
Unemployed – Retired	42.0	31.3		
Unemployed – volunteer, student, unemployed	31.4	19.3		
RISK FACTORS/PRE-STROKE STATUS				
Atrial Fibrillation, %	10.1	2.1	0.004	
Diabetes Mellitus, %	17.1	16.6	0.8	
Current Tobacco Smoker, %	33.4	38.2	0.13	
Hypercholesterolemia, %	19.5	23.8	0.4	
Hypertension, %	94.5	98.0	0.055	
Previous stroke, %	18.6	9.4	0.002	
Pre-Stroke Condition, %				
Independent	92.4	95.1	0.14	
Dependent at home or in facility	7.7	4.8		
Pre-Stroke Rankin Scale, %			0.048	0.9
No disability	76.9	73.5		
No significant disability	10.7	17.4		
Slight disability	3.0	2.1		
Moderate disability, able to walk	4.6	2.2		
Moderate disability, unable to walk	2.6	1.6		
Severe disability	2.2	3.2		
CLINICAL CHARACTERISTICS AT PRESENTATION				
Disturbed Consciousness, %	30.3	60.4	<0.001	
Speech Disturbances, %	59.7	70.3	0.005	
Weakness, %	93.1	80.5	<0.001	
Systolic blood pressure, mmHg, mean	154.9	171.3	<0.001	
Diastolic blood pressure, mmHg, mean	86.2	92.3	<0.001	
Glucose level, mmol/L, mean	7.3	8.0	0.055	
Cholesterol level, mmol/L, mean	5.0	4.7	0.021	
PROCESSES OF CARE^+^				
Ward, %			<0.001	
Intensive Care	11.9	43.5		
Cardiology	74.6	52.2		
Other	13.4	4.3		
Occupational therapist, %	10.1	2.4	0.013	
Physical therapist, %	2.7	1.0	0.3	
Speech therapist, %	1.7	0.0	<0.001	
Swallowing assessment, %	15.5	5.5	0.006	
Thrombosis, %	1.6	1.0	0.6	
Carotid ultrasound, %	1.7	0.0	<0.001	
CT Scan	99.6	99.6	0.7	
ECG, %	89.1	88.3	0.8	
LP, %	0.3	0.0	<0.001	
MRI, %	2.4	0.4	0.5	
Pneumonia, %	8.2	12.4	0.13	

*P-value for F-test of univariate model with single categorical variable with n-1 degrees of freedom where n=number of categories.

^+^Excludes patients who died in hospital and patients who were discharged to die at home.

Processes of care, as measured in patients that did not die in hospital nor were discharged home to die, also differed by stroke type (Table 1). Patients with ischemic stroke were more likely to have been treated in a cardiology ward, more likely to have had occupational therapy, speech therapy and swallowing assessment. The vast majority of both ischemic and hemorrhagic strokes had a CT scan and an electrocardiogram (ECG). Few patients had evaluation by carotid ultrasound (1.7% of ischemic stroke), lumbar puncture (LP) or magnetic resonance imaging (MRI).

A total of 496 (65.8%) patients were alive at and discharged from the hospital; 49 (6.5%) died in the hospital; and an additional 209 (27.7%) were in grave condition or dying and were released to die at home, as requested by the patients themselves or by their family members. Of note, all patients “discharged to die” were classified as having mRS = 5 (severe disability) at the time of discharge and all but 2 of the patients died later the same day as discharge (the other 2 dying 5 and 17 days after discharge). At 28-day follow-up, 261 (36.4%) patients had died, 456 (63.6%) were still alive, and 37 were either lost to follow-up or refused to be re-contacted. Outcomes for those lost to follow-up were imputed for further analyses [3]. The 28-day crude mortality in our study population was 20.3 % for ischemic stroke and 51.0% for hemorrhages resulting in an overall 37%.

Table 2 summarizes differences in medications and outcomes between those with ischemic and hemorrhagic stroke after all missing data were multiply imputed. At discharge, diabetes medication, antiplatelet medication and cholesterol lowering medication were more commonly provided to patients with ischemic strokes than those with hemorrhagic strokes. Combined, at discharge, 51.8% of ischemic stroke patients were treated with either anticoagulants or antiplatelet agents.

**Table 2:** Medications given to patients alive at discharge*, and outcomes for all patients, according to stroke type

OUTCOMES*	**Ischemic**	**Hemorrhagic**	p-value**	**Trend p-value**
N (%)	328	366	-	-
Modified Rankin score at discharge, %			0.2	<0.001
No disability	0.9	0.2		
No significant disability	8.5	3.4		
Slight disability	10.7	6.2		
Moderate disability, able to walk	21.8	12.0		
Moderate disability, unable to walk	17.0	11.1		
Severe disability	37.7	57.8		
Dead	3.5	9.4		
Modified Rankin score at discharge, mean	3.81	4.78	<0.001	
Discharge status, %			<0.001	
Alive	81.2	50.5		
Dead	3.6	9.4		
Discharged to die	15.3	40.1		
28-day Mortality	20.3	51.0	<0.001	
MEDICATIONS AT DISCHARGE^+^				
Anticoagulants in-hospital, %	2.3	0.5	0.2	
Antidiabetics in-hospital, %	8.5	4.0	0.10	
Antiplatelets in-hospital, %	49.5	1.5	<0.001	
Cholesterol lowering medications in-hospital, %	38.9	23.6	0.001	
Antihypertensives, %	55.7	77.5	<0.001	

*Missing values multiply imputed.

**P-value for F-test of univariate model with single categorical variable with n-1 degrees of freedom where n=number of categories.

^+^Excludes patients who died in hospital and patients discharged to die at home.

Those who died by 28-day follow-up differed significantly by univariate analysis on several characteristics from those who were still alive (Table 3). Pre-stroke patient characteristics associated with death by 28 days included male gender, employment status, patients who were physically dependent or with greater disability prior to the stroke and those with diabetes, hypercholesterolemia, and current tobacco use. Markers of stroke severity were strongly associated with 28-day mortality and included hemorrhagic stroke type and initial symptoms such as disturbed consciousness and speech disturbances. Weakness as an initial symptom was less common in patients that died (74.1% vs. 93.6%). Initial biological measures associated with 28-day mortality included both systolic and diastolic blood pressure, glucose and cholesterol levels. Services and procedures associated with 28-day mortality included receiving care in the ICU, less use of ECG, slightly more LPs (1.2% vs. 0.0%) and less use of antiplatelet agents.

**Table 3:** Characteristics of 754 stroke patients admitted to Da Nang Hospital according to 28-day mortality

Characteristics*	**Died**	**Alive**	p-value**	**Trend p-value**
N (%)	261	456	-	-
DEMOGRAPHICS				
Male, %	67.0	57.9	0.014	
Age, years, %			0.13	0.5
1st Quartile (31–53)	27.4	21.5		
2nd Quartile (53–65)	22.2	27.7		
3rd Quartile (66–76)	23.1	25.1		
4th Quartile (77+)	27.3	25.7		
Age, years, mean	64.5	65.3		0.5
Education, %			0.14	0.8
<Primary school	31.3	33.4		
Completed primary and secondary school	50.6	44.4		
≥High school	18.1	22.3		
Employment, %			0.01	
Currently employed	42.2	36.1		
Unemployed - Retired	39.5	34.9		
Unemployed - volunteer, student, unemployed	18.2	29.0		
RISK FACTORS/PRE-STROKE STATUS				
Atrial Fibrillation, %	7.2	5.5	0.4	
Diabetes Mellitus, %	31.0	9.0	<0.001	
Current Tobacco Smoker, %	40.6	33.4	0.038	
Hypercholesterolemia, %	36.0	14.0	<0.001	
Hypertension, %	96.1	96.5	0.6	
Previous stroke, %	12.8	14.7	0.5	
Pre-Stroke Condition, %				
Independent	88.7	96.5	<0.001	
Dependent at home or in facility	11.3	3.5		
Prior Rankin Scale, %			<0.001	<0.001
No disability	54.4	86.5		
No significant disability	28.3	6.3		
Slight disability	4.2	1.7		
Moderate disability, able to walk	4.2	2.9		
Moderate disability, unable to walk	3.4	1.5		
Severe disability	5.7	1.1		
CLINICAL CHARACTERISTICS				
Stroke Subtype, %				
Ischemic	26.4	52.2		
Hemorrhagic	72.0	36.8		
Disturbed Consciousness, %	86.3	23.2		
Speech Disturbances, %	88.5	52.2	<0.001	
Weakness, %	74.1	93.6	<0.001	
Systolic blood pressure, mmHg, mean	172.6	158.6	<0.001	
Diastolic blood pressure, mmHg, mean	92.5	87.5	<0.001	
Glucose level, mmol/L, mean SD	10.3	6.2	<0.001	
Cholesterol level, mmol/L, mean SD	4.7	5.0	0.15	
PROCESSES OF CARE^+^			<0.001	
Ward, %				
Intensive Care	77.2	1.0		
Cardiology	20.0	87.0		
Other	2.7	12.0		

*Missing values multiply imputed.

**P-value for F-test of univariate model with single categorical variable with n-1 degrees of freedom where n=number of categories.

^+^Excludes patients who died in hospital and patients who were discharged to die at home.

Predictors of 28-day mortality that remained independently significant in multivariate models included hemorrhagic stroke type, worse pre-stroke mRS, disturbed consciousness, absence of observed weakness at presentation, higher diastolic blood pressure, higher glucose levels, current tobacco smoking and history of hypercholesterolemia (Table 4). The most powerful independent predictor was the presence of disturbed consciousness with a greater than 3-fold increase in the risk of 28-day mortality. Patients with hemorrhagic stroke had a 49% increased risk after controlling for all other characteristics. Table 4 also presents estimates from models in which missing data were not imputed. The estimates of the non-imputed and multiply imputed models were, for the most part, similar in the direction, estimates, and the significance of predictors with the exception of severe disability on prior mRS. The relative risk of mortality for severe disability of the non-imputed model was 0.60 (95% CI: 0.21, 1.75) compared to 1.64 (95% CI; 1.23, 2.18) using multiple imputation.

**Table 4:** Independent predictors of 28-day mortality among 754 stroke patients

	**No imputation**		**Trend test p-value**	Multiply imputed*		**Trend test p-value**
	RR**	**95% CI**		RR**	**95% CI**	
**Male**	**1.59**	**(1.18, 2.14)**		**1.28**	**(1.07, 1.54)**	
**Age**			**0.017**			**0.2**
**1st Quartile (31–53)**	**1.00**	**Reference**		**1.00**	**Reference**	
**2nd Quartile (53–65)**	**0.92**	**(0.66, 1.28)**		**0.80**	**(0.66, 0.96)**	
**3rd Quartile (66–76)**	**1.06**	**(0.73, 1.55)**		**0.97**	**(0.76, 1.24)**	
**4th Quartile (77+)**	**1.60**	**(1.09, 2.37)**		**1.08**	**(0.85, 1.35)**	
**Education**			**0.025**			**0.018**
**<Primary school**	**1.00**	**Reference**		**1.00**	**Reference**	
**Completed secondary school**	**0.87**	**(0.64, 117)**		**1.01**	**(0.79, 1.28)**	
**≥High school**	**0.57**	**(0.39, 0.85)**		**0.71**	**(0.57, 0.95)**	
**Stroke subtype**						
**Ischemic**	**1.00**	**Reference**		**1.00**	**Reference**	
**Hemorrhagic**	**1.41**	**(1.03, 1.91)**		**1.49**	**(1.22, 1.82)**	
**Prior Ranking score**			**0.18**			**<0.001**
**No disability**	**1.00**	**Reference**		**1.00**	**Reference**	
**No significant disability**	**1.67**	**(1.28, 2.19)**		**1.86**	**(1.56, 2.21)**	
**Slight disability**	**2.56**	**(1.48, 4.44)**		**2.40**	**(1.67, 3.45)**	
**Moderate disability, able to walk**	**2.36**	**(0.99, 5.66)**		**2.35**	**(1.50, 3.68)**	
**Moderate disability, unable to walk**	**1.74**	**(0.68, 4.48)**		**2.28**	**(1.42, 3.68)**	
**Severe disability**	**0.60**	**(0.21, 1.75)**		**1.64**	**(1.23, 2.18)**	
**Disturbed consciousness**	**4.65**	**(2.88, 7.50)**		**3.72**	**(2.62, 5.73)**	
**Weakness**	**0.69**	**(0.53, 0.92)**		**0.66**	**(0.55, 0.78)**	
**Speech disturbance**	**0.78**	**(0.47, 1.28)**		**1.54**	**(1.10, 2.16)**	
**Diastolic blood pressure, per 10 mmHg**	**1.17**	**(1.09, 1.24)**		**1.05**	**(1.10, 2.16)**	
**Glucose level, per 1 mmol/L**	**1.02**	**(1.01, 1.03)**		**1.02**	**(1.01, 1.03)**	
**Hypercholesterolemia**	**1.42**	**(1.15, 1.77)**		**1.33**	**(1.12, 1.55)**	
**Prior stroke**	**0.51**	**(0.27, 0.98)**		**0.62**	**(0.45, 0.84)**	

*multiple imputation resulted in 10 datasets that were combined to produce results.

**adjusted for all other variables.

## Discussion

In this hospital-based prospective cohort study from Da Nang Hospital in Vietnam, we enrolled 754 stroke patients over one year and observed a 28-day crude mortality of 37%. The proportion of confirmed hemorrhagic strokes was nearly 50%. A number of factors were independently associated with 28-day mortality, the two strongest of which were depressed level of consciousness on presentation and hemorrhagic stroke type. Also, a number of observations about processes of care were worthy of note in that they are different than in many western medical systems and, in some cases, may represent opportunities for evidence-based interventions to improve outcomes.

The 37% 28-day overall mortality observed in this cohort is similar to the mean overall early mortality of 35.7% in population-based studies of strokes from low and middle-income countries during the 1980’s, but higher than the 2000–2008 26.6% early mortality estimate from low and middle-income countries and the 19.8% from high-income countries [3]. The stroke type specific mortality (20.3 % for ischemic, 51% for hemorrhages) can be compared to the population-based early mortality rates in low and middle-income countries for ischemic stroke at 16.7% (range 13-19%) and ICH at 38.7% (range 30-48%) from 2000–2008 [3]. Our Da Nang hospital-based stroke type specific estimates of mortality are slightly higher in both stroke types, and show the expected higher mortality of patients with hemorrhages over those with ischemic strokes; thus a good portion of the overall increased mortality in our cohort is due to the higher mortality among patients with hemorrhagic stroke. One of the population-based studies with the highest mortality estimate was from Tbilisi, Georgia (classified similar to Vietnam as a lower middle income country by the World Bank [11]), included patients from 2001–2003 and found ischemic stroke early mortality was 19.2% and ICH 48.4% [12]. These high estimates were, in part, blamed on “lack of an organized stroke service with specialized stroke units,” which may also be a factor in Vietnam. Hospital-based mortality rates from the United States (US) can be found in reports based on the Get-with-the-Guidelines (GWTG) Stroke quality improvement registry. The in-hospital mortality or discharge to hospice proportions by stroke type in the first million patients was 9.1% for ischemic stroke and 30.7% for ICH, again lower than the 28-day mortality estimate in our Vietnam cohort [13]. In the fully ascertained population-based samples used for comparison [3], patients with less severe strokes are included but are less likely to be represented in hospital-based cohorts because they may simply stay home or see a health care provider without hospitalization. This question of who gets hospitalized may lead to a referral bias in our hospital-based cohort with selection of more severe cases and may be a partial explanation for the Vietnam cohort’s higher mortality compared to the population-based studies. Also, the patients included in the GWTG-Stroke registry are those for whom the stroke ICD-9 code is in the primary position [14]. In an earlier report-based on administrative stroke data, we showed that patients whose ischemic stroke ICD-9 codes were in non-primary positions (who would thus be excluded from GWTG-Stroke) were much more likely to die by 30 days, 30%, vs. 12.6% for those with a primary position ischemic stroke ICD-9 code [15]. These GWTG hospital discharge mortality data thus likely underestimate true early mortality in all hospitalized stroke patients.

A number of studies have shown that the proportion of stroke due to ICH is higher in Asian countries [16]. Older studies from Japan supported a higher proportion of ICH, though as hypertension treatment has increased, this excess has become less apparent [17]. A review of stroke epidemiology from China notes ICH proportions from more recent studies of 19-48%. One study spanning the years 1986–2000 reported an ICH proportion as high as 55% [18,19]. The distribution of stroke types also varies by income level, with double the proportion of ICH documented in middle and low income countries (22%) compared to high income countries (11%) [3]. Our data from Da Nang, Vietnam is consistent with this literature; we observed that nearly 50% of hospitalized stroke patients had ICH. This is likely related to a high proportion of untreated hypertension in Vietnam. In a parallel study in Da Nang, we found 32.9% of community adults over age 35 to have hypertension, with more than two-thirds of participants unaware of their condition [20]. Some of this high proportion of hemorrhagic stroke may also have been related to structural abnormalities such as vascular malformations or tumors, but neither contrast CT nor CT angiography were part of the local standard of care, so these conditions were not identified.

The independent predictors of 28-day mortality in our stroke cohort are broadly consistent with many previous studies. The most powerful single patient characteristic was “disturbed level of consciousness” at the time admission to the hospital, a marker of a very severe stroke; speech disturbance was also associated with mortality, but weakness seemed to have a paradoxical inverse association. This latter finding may represent an ascertainment bias, whereby patients with the most severe strokes (and likely depressed level of consciousness), were not able to cooperate with a neurologic exam enough to demonstrate weakness. In a recent study of hospitalized stroke patients from Fortaleza, Brazil, depressed level of consciousness was also the strongest independent predictor or poor outcome at discharge, other significant predictors being age and pre-stroke disability [21]. Stroke severity is consistently the most powerful predictor of stroke outcome, as has been known scientifically for decades and by clinicians since ancient times [22]. Pre-stroke disability is consistently reported as an important prognostic indicator and was also found to be the case in our patients from Vietnam.

A Stroke Unit is defined as “a discrete area in the hospital that is staffed by a specialist stroke multidisciplinary team. It has access to equipment for monitoring and rehabilitating patients. Regular multidisciplinary team meetings occur for goal setting.” [23]. Stroke Units are a highly evidence-based way to improve outcomes after stroke [24]. Systematic meta-analyses show an estimated 14% reduction in the odds of death at ~1 year follow up and an 18% reduction in the odds of death or dependency [24]. The experience with Stroke Unit care in Vietnam is limited, with fewer than 10 Stroke Units reported in the country in 2007 [25]. A major limitation to the formation of more stroke units, and potentially improved outcomes, may be the limited availability of stroke specialists; while ICU physicians, cardiologists and general internal medicine doctors traditionally care for these patients, stroke does not appear to be a special focus. These limitations in capacity, resources, infrastructure and training likely contribute to the high mortality. The Da Nang Hospital lacks a formal Stroke Unit, but virtually all stroke patients are admitted to either the ICU or the Cardiology unit. Such a consistent geographic placement of stroke patients suggests that additional training of a focused group of health care providers could lead to “stroke unit” like conditions and greater and more consistent implementation of internationally recognized stroke standards of care.

Carotid endarterectomy (CEA) is a highly evidence-based intervention for prevention of stroke due to high grade symptomatic stenosis [26]. Carotid ultrasound has been identified as a quality indicator for acute ischemic stroke care, in individuals who would otherwise be eligible for CEA [27]. The application of carotid ultrasound was low in our ischemic stroke subgroup (1.7%), suggesting a lack of resources for more routine use. Also factoring into this low rate is the lack of a surgeon in Da Nang who can perform CEA, but patients can be transferred to Ha Noi or Ho Chi Minh City if CEA is needed. Similarly, the Fortaleza, Brazil study suggested many care resources are not present in more remote hospitals [21]. A commentary on the Brazil study suggested there is utility in the reporting of modest results, as they reveal the possibility of improving quality of care as a result [28].

The use of antithrombotic medications (antiplatelet or anticoagulation medications) during hospitalization and prescribed at the time of hospital discharge is a widely accepted standard of care in western countries for ischemic stroke (28). Despite this standard, and even after limiting our assessment to ischemic stroke patients that neither died in hospital nor were discharged home to die, anti-thrombotics were only used in 52% of ischemic stroke patients. This low proportion may in part be appropriate, in that there are a number of valid reasons why some patients may not be able to take antithrombotic agents (e.g. allergy to aspirin, or history of severe bleeding condition), but such exclusions would not likely increase compliance to 100%. Consistent with this suboptimal rate of antithrombotic use are the results from the 2011 PURE study showing use of simple inexpensive medications for patients with a history of cardiovascular disease, including stroke, is low and proportional to country income level [29].

A culturally unique observation was the large proportion of patients “discharged home to die,” as occurred in 15% of ischemic stroke cases and 40% of ICH (Table 2). The general Vietnamese belief holds that people should die at home, where they spent most of their lives, and with family members around and caring for them. If the doctor thinks that the patient has little chance to survive they will inform the family of the situation, and the family makes the final decision to take the patient home [30]. A series of rituals are performed starting at the time of eminent death to one year after death. As described above, virtually all of the patients discharged to die at home did so within the first day of leaving the hospital. Although Vietnamese hospitals do not currently have formal do-not-resuscitate orders in place, this cultural practice mirrors withdrawal of life sustaining interventions (WLSI) and do-not-resuscitate orders (DNRs) in the US, which account for the majority of deaths in ICH [31][32]. In fact, WLSI and DNR orders doubles the risk of death even after adjusting for clinical and radiographic characteristics [33]. A “self-fulfilling prophecy” may exist if patients with particular clinical or radiographic characteristics are presumed to have a poor outcome, and based on this presumption, life-sustaining interventions are withdrawn or DNR orders are established [34,35]. These early care limitations have been shown to independently predict mortality in stroke patients [31].

The strengths of our study are numerous. Patient-level detailed data were collected in a prospective fashion from all stroke patients presenting to Da Nang Hospital, which supplies the vast majority of health care in the area. Only a few patients were lost to follow-up, only a moderate amount of data points were missing, and the use of imputation allowed for inclusion of all patients in analyses. The high level of 28-day follow-up we obtained contrasts to difficulties obtaining these data in previous WHO Stroke STEPS endeavors [36]. We have demonstrated that collaboration between stroke caregivers in high and low-income countries is feasible and can add to the sparse literature on the processes and outcomes of care in resource-poor settings. Gaps in evidenced-based, and often inexpensive, care for stroke patients were identified and will serve as targets for future interventions that may be generalizable to other resource-poor stroke care settings.

Limitations of our study include the lack of true population-based review of stroke cases; patients with acute stroke not admitted to the hospital were not captured, and so our sample may not be representative of the true stroke burden in Da Nang. Furthermore, we did not conduct a complete assessment of all potentially important risk factors for stroke. Alcohol consumption, for instance, has been shown in several studies to be associated with the risk of hemorrhagic stroke [37]. Other important risk factors that can be assessed in future studies include physical activity and body mass index. Greater detail about stroke risk factors prior to stroke presentation, and more detail on stroke etiology may allow a better understanding of culturally relevant approaches to stroke prevention. Also, stroke outcomes are often best assessed 3–6 months after stroke, once much of the recovery has occurred.

## Conclusions

The global burden of stroke in Vietnam, similar to other low- and middle-resource counties, is taking a disproportionate toll on its people. This cohort study of 754 stroke patients from Da Nang hospital highlights the high early mortality and disability due in part to the large proportion of hemorrhagic strokes. Access to evidence-based standards of care was limited by lack of local resources and local evidence. As a result, opportunities for simple, inexpensive interventions to improve outcomes or reduce recurrent stroke have been identified. In future work, we hope to facilitate implementation of widely accepted, and locally feasible, standards of care that have the potential to reduce the burden of stroke in Vietnam and other nations facing the health transition.

## Abbreviations

CEA: Carotid endarterectomy; CT: Computed tomography; ECG: Electrocardiogram; GWTG: Get-with-the-Guidelines; ICH: Intracerebral hemorrhage; ICU: Intensive care unit; LP: Lumbar puncture; mRS: Modified Rankin Scale; RR: Relative risk; SAH: Subarachnoid hemorrhage; US: United States; WHO: World Health Organization.

## Competing interests

Coauthors have no competing interests to report.

## Authors’ contributions

ALF was the principal investigator of the main research project and was primarily responsible for the administration and conduct of the study. She led the team in formulating the research questions and producing the survey instrument. She provided training to field workers and in epidemiology and research method to the local researchers. DLT provided clinical training in stroke epidemiology to local stroke providers. He contributed to the conception and design of the study, interpretation of the data, drafted the manuscript as the primary author. KAL was the project coordinator and primary liaison with the local research team. QVN was the local project coordinator. CHP was the primary local collaborator who contributed to the design of the study and managed local project coordinators. KAL and QVN participated in formulating research questions, producing and translating survey instruments, and training field workers. They served as conduits with local authorities regarding study design and procedures. They implemented and supervised the study, visited field sites, and critically revised the manuscript. TTV developed and managed the study database. TTV and QVN double-entered and checked data for quality control. TGNT provided methodological expertise and conducted data cleaning and analyses. She also provided training to field workers in epidemiologic and analytic methods as well as critically revised the manuscript. WL contributed to the conception and design of the study, interpretation of the data, critically revised the manuscript. All authors read and approved the final manuscript.

## Pre-publication history

The pre-publication history for this paper can be accessed here:

http://www.biomedcentral.com/1471-2377/12/150/prepub
